# Molecular mechanisms behind mRNA localization in axons

**DOI:** 10.1098/rsob.200177

**Published:** 2020-09-23

**Authors:** Benita Turner-Bridger, Cinzia Caterino, Jean-Michel Cioni

**Affiliations:** 1 Department of Physiology, Development and Neuroscience, University of Cambridge, Downing Street, Cambridge, UK; 2 Division of Neuroscience, IRCCS San Raffaele Scientific Institute, Via Olgettina 60, 20132 Milan, Italy

**Keywords:** mRNA trafficking, RNA-binding proteins, axon, neuron, local translation

## Abstract

Messenger RNA (mRNA) localization allows spatiotemporal regulation of the proteome at the subcellular level. This is observed in the axons of neurons, where mRNA localization is involved in regulating neuronal development and function by orchestrating rapid adaptive responses to extracellular cues and the maintenance of axonal homeostasis through local translation. Here, we provide an overview of the key findings that have broadened our knowledge regarding how specific mRNAs are trafficked and localize to axons. In particular, we review transcriptomic studies investigating mRNA content in axons and the molecular principles underpinning how these mRNAs arrived there, including cis-acting mRNA sequences and trans-acting proteins playing a role. Further, we discuss evidence that links defective axonal mRNA localization and pathological outcomes.

## 1. Introduction

Neurons have the ability to span huge distances across the body. The estimated average cumulative length of a human forebrain cholinergic axon is approximately 100 m [1], while the dendritic branches of cat spinal alpha-motor neurons have an average combined length of nearly 5 mm [2]. This complex morphology permits the precise connectivity needed to relay and process information across the nervous system. But while these figures illustrate the geometric diversity of different neuron types and the remarkable lengths of their cytoplasmic processes, they also present a dilemma. With long axonal and dendritic processes, how do neurons quickly respond to signals with high spatial precision when the source of the signal can sometimes be metres away from the cell body? One solution is to traffic particular sets of messenger RNAs (mRNAs) into neuronal processes, where they can then be locally translated under basal conditions and in response to particular cues. This approach of localizing mRNAs to axonal and dendritic processes offers several advantages. First, because mRNAs are often transported in a translationally silent state, it allows proteins to act at their site of synthesis, facilitating the specific protein interactions that are needed locally (such as complex formation), and preventing aberrant protein expression and functioning that could be deleterious at other cellular regions. Second, since many proteins—typically thousands [3,4]—are translated from a single mRNA, it provides an energy efficient means to localize numerous proteins by transporting the mRNA molecule that encodes them. Lastly, mRNA localization provides polarized cells with important degrees of subcellular autonomy. Axons and dendrites have different functions, and each needs to respond to different environmental signals to the cell body. By localizing specific subsets of mRNAs to particular subcellular locations, these regions subsequently have the ability to spatiotemporally regulate their own proteome both in response to local demands and to extracellular cues.

The role of mRNA localization in neuronal axons was once a controversial topic. Early studies identified polysomes and protein synthesis apparatus in dendrites [5,6] but did not detect axonal polysomes, nor the presence of Golgi apparatus and rough endoplasmic reticulum (RER) needed for processing newly synthesized proteins [5,7,8]. Since then, however, technological advances have enabled direct visualization of axon mRNA translation [9], actively translating ribosomes have been isolated from axons [10], and components of both Golgi apparatus and RER have been shown to be distributed along axons [11]. For the latter, though, a dearth of evidence for the classical structure and functioning of RER and Golgi apparatus in axons has led to the suggestion of non-canonical pathways for processing newly synthesized transmembrane and secreted proteins in axons [12]. In addition, monosomes (single ribosomes which are abundant in axons) have also been demonstrated to actively translate [13,14]. The last 25 years has witnessed an upsurge of studies outlining the many important roles that axonal mRNA translation plays. During the development of neural connectivity, local translation has been implicated in axon outgrowth, navigation, branching and synaptogenesis (reviewed in [15]). In addition, axonal protein synthesis continues into adulthood, where local mRNA translation is not only implicated in regeneration [16], but also in presynaptic plasticity as well as synapse maintenance [17–20].

In this review, we will focus on one specific (and little understood) aspect of regulating local mRNA translation in axons: the localization of the mRNA itself. What are the mRNAs that are present in axons and how do they move? Which features allow mRNAs to selectively reach the axon tip? Could defects in axonal mRNA localization underlie the pathological mechanisms behind neurological disorders? A wealth of studies has begun to address these important questions and paved the way for future progress in the field.

## 2. Transcriptome analysis: a general view on axonal mRNA localization

Thousands of mRNAs have been demonstrated to localize to axons, forming functionally relevant mRNA libraries that are separate in composition to the soma, which suggests the existence of molecular mechanisms for selective axonal targeting [21–25]. Repeatedly, it has been shown that axons are enriched with groups of mRNAs that have relevant functions in known axonal processes, like those encoding proteins associated with translational regulation, mitochondria functions and intracellular transport [21–26]. Within the axons themselves, different mRNAs have different localization patterns—some are enriched in the axon shaft (e.g. mRNAs encoding proteins involved in protein trafficking and folding), and others are specifically enriched in the distal growth cone (e.g. mRNAs encoding proteins involved in cytoskeletal regulation) [21]. By contrast, certain mRNAs, such as *map2* and *γ*-actin, are present in the cell body but are selectively excluded from axons of particular neurons [27,28]. Several of these axon-enriched mRNA categories have recently been shown to comprise a conserved core neurite (dendrite and axon) transcriptome. These include mRNAs coding for translational machinery, cytoskeletal proteins as well as mitochondrial proteins, and indicate that certain mRNAs are constitutively localized to neuronal projections regardless of projection type and species [29].

### 2.1. The changing axonal transcriptome

Axonal transcriptomes have been shown to vary to some extent between neuron types. A recent article comparing transcriptome datasets from dorsal root ganglion (DRG) and motor neuron axons revealed that two-thirds of axonally enriched mRNAs are shared. Thus, while there is a good fraction of overlap, a degree of discrepancy also exists that indicates specialization in axonal transcriptomes between neurons [30]. These differentially enriched mRNAs include those with gene ontology (GO) functions in receptor activity, chemotaxis and synapse assembly, and may consequently entail motor neuron-specific aspects of development and functioning [30]. Indeed, another example of a neuron-type transcriptome difference is exemplified by actin mRNA isoforms. Here, the axonal compartment of neurons has long been thought to only contain the *β-actin* mRNA isoform [28,31]. However, it has recently been reported that *α-, β-* and *γ*-actin mRNAs are present in motor neuron axons, their local translation playing different roles in axon branching and growth [32]. Although a fascinating question, it remains to be seen how different local pools of mRNAs in axons might help confer specific functions related to neuron-type through local translation. Some specializations are, however, quite self-explanatory; an example being the localization of mRNAs encoding olfactory receptors in olfactory neuron axons [33,34].

As the neuron develops, changes in the axonal transcriptome have been reported to occur; these are thought to support the specific needs of the axon at different points in time. For instance, while some transcripts are constitutively enriched in both embryonic and adult DRG axons (e.g. mRNAs linked to translation and mitochondria function), others, such as mRNAs encoding cytoskeletal regulators, cell cycle and intracellular transport proteins, are specifically enriched in embryonic axons, while adult DRG axons are enriched for mRNAs associated with inflammation and the immune response [24]. Throughout development itself, retinal ganglion cell (RGC) axons change their transcriptome. Young growth cones are particularly enriched with mRNAs coding for regulators of protein synthesis, and as development progresses, there is an upregulation in mRNAs encoding proteins involved in processes such as signalling and metabolism in older growth cones [21]. These developmental changes in axonal mRNA repertoires are reflected in the translatome of mouse RGCs *in vivo* [10]. Interestingly, however, developmental changes in the axonal transcriptome precede their translation, indicating that the mRNAs are initially stored in a translationally silent state before becoming unmasked at a later time point [10].

The axonal transcriptome can also be modified by the extracellular environment. One classic example is the observation that axon guidance cues and growth factors like Neurotrophin-1, BDNF, Netrin-1 and Sonic Hedgehog, can stimulate increased growth cone localization of *β-actin* mRNA [35–39]. When BDNF and Netrin-1 are introduced as a gradient, *β-actin* mRNA translocates to the growth cone periphery on the near side of the attractive guidance cue stimulation, where asymmetric β-actin protein synthesis is thought to promote preferential actin filament assembly on one side and thus growth cone turning [38,39]. While a comprehensive analysis of modulated axonal mRNA repertoires in response to extrinsic cues is missing, extracellular signals have been shown to regulate axonal localization of many mRNAs encoding proteins such as enzymes, antiapoptotic factors, endoplasmic reticulum chaperones and cytoskeletal regulators [25,31,40,41]. Recently, a proteomic study revealed how different extracellular cues alter the repertoire of newly synthesized proteins in axons at different time points after cue application [42]. This study raises the question as to what extent altered mRNA localization is responsible for cue-induced proteome remodelling in axons. Regulation of mRNA localization has been shown to be the main contributing factor for protein localization in neurites [43]. However, extracellular signals can also alter protein synthesis patterns in axon terminals by enhanced translation of mRNAs independent of changes in mRNA localization [44]. Coordinate investigation into axonal mRNA transcriptome and protein synthesis changes in response to extrinsic signals will therefore be an important and fascinating question for the future.

### 2.2. Insights into axonal mRNA content *in vivo*

While many axonal transcriptome studies have been performed *in vitro*, recent work has also provided important insights into axonal mRNA localization *in vivo*. For example, by developing a method to sort growth cones of mouse callosal projection neurons *in vivo*, Poulopoulos *et al*. [45] discovered an enrichment of transcripts containing 5′ terminal oligopyrimidine (TOP) motifs (including mRNAs encoding translational machinery). Interestingly, the translation of TOP-containing transcripts is dependent on the mammalian target of rapamycin (mTOR), which is enriched in the callosal projection neuron growth cones (along with other proteins in the mTOR pathway) and required for axon growth. This focal distribution in growth cones suggests a mechanism for precisely controlling the localization of these mRNAs and their translation in axons *in vivo* [45]. In another recent study, Hafner *et al.* [44] employed ‘fluorescence-activated synaptosome sorting (FASS)’ in VGLUT1^+venus^ knock-in mice to specifically isolate presynaptic compartments. Here, the authors demonstrated that *in vivo* adult excitatory axon terminals are enriched with mRNAs encoding presynaptic proteins, such as those involved in presynaptic vesicle release [44]. These results are in accordance with an *in vivo* study in *Caenorhabditis elegans* that further confirmed an axonal enrichment of mRNAs with specialized presynaptic functions, as well as revealing the presence of mRNAs encoding RNA-binding proteins (RBPs), including the RBP Pumilio which plays a role in regulating associative memory formation [46]. Together, these transcriptome data highlight the emerging role of axonal mRNA localization in regulating presynaptic development and functioning *in vivo* [17,47,48].

## 3. RNA-binding proteins: the cornerstones of axonal mRNA localization

Transcriptomic studies have thus revealed that particular sets of mRNAs are enriched in axons, but how are they selectively targeted to axons in the first place? The answer is thought to lie with RBPs.

### 3.1. mRNA regulation by RBPs (a brief overview)

**Figure 1.**
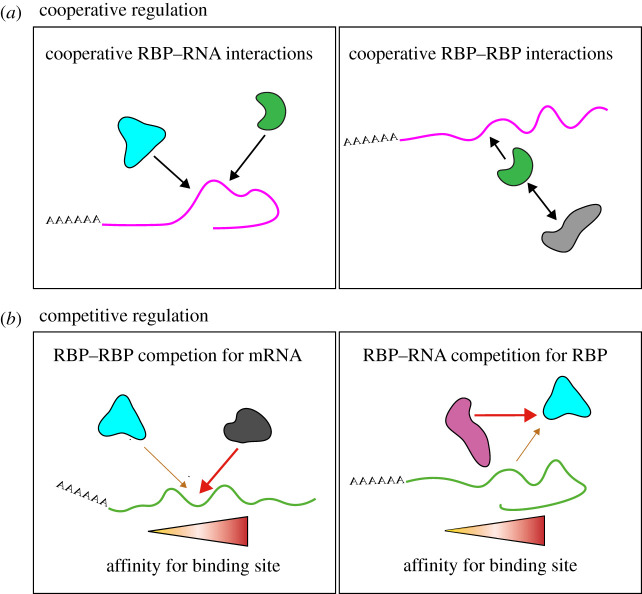
Molecular interplay between RNA-binding proteins and mRNAs. Schematic of the molecular mechanisms by which RBPs can regulate mRNA localization. (*a*) Cooperative regulation. Different RBPs bind to the same target mRNA and promote its localization (left panel). Alternatively, an RBP can recruit additional RBPs to the mRNP through protein–protein interactions and together drive its localization (right panel). (*b*) Competitive regulation. RBPs with distinct localization compete for the binding to the target mRNA with different affinities. The localization of the mRNA is thus controlled by the relative abundance of the RBPs (left panel). RBP–RBP interactions prevent the binding to a target mRNA and thus its subcellular localization (right panel).

Studies in a variety of cell types and organisms have shown that mRNAs do not travel alone. Instead, they are processed, trafficked and ultimately degraded as part of complexes, collectively named ‘messenger ribonucleoprotein particles,’ or ‘mRNPs’. Such complexes are highly variable in composition; made up of many different proteins, noncoding RNAs, ions and small organic molecules which can remain permanently associated or be dynamically exchanged as the mRNP is remodelled throughout the mRNA's lifetime [49,50]. Here, RBPs facilitate the selective recruitment of mRNAs into mRNPs. In the nucleus, mRNAs are co- and post-transcriptionally recognized by RBPs; an interaction that subsequently governs each step of their maturation and ensuing nuclear export [51,52]. Both during and after nuclear export, mRNPs are further remodelled. For example, the nuclear export receptor complexes are displaced from the mRNP at the cytoplasmic face of nuclear pores in an ATP-dependent manner [53]. In addition, the mRNP is now exposed to new cytoplasmic RBPs, which can, for instance, promote cytoplasmic mRNP transport along cytoskeletal tracks (see §5). A restricted number of RNA-binding domains have been characterized so far, including the K homology (KH) domain, zinc fingers (Znf) and the RNA recognition motif (RRM) [51]. These classical globular RNA-binding domains form structures that recognize particular sequences within an mRNA transcript through shape complementarity and interactions with nucleotide bases [54]. Further, the binding affinity between mRNA and RBP can rely on single or multiple domains present in an RBP [54,55]. Aside from classical RNA-binding domains, RBPs can interact with mRNAs through unconventional or uncharacterized RNA-binding sites, including located within certain intrinsically disordered regions [56]. Each RBP associates with a particular subset of mRNAs, and individual mRNAs have the ability to bind to multiple RBPs. Ultimately, precise control of the mRNP's molecular signature is thought to be essential for targeting to a subcellular location and is achieved through multiple modes of action by different RBPs (figure 1*a*,*b*). However, how this is regulated in neurons to control axonal mRNA localization is still unclear.

### 3.2. RBPs regulating mRNA localization in axons

One of the first RBPs discovered to regulate axonal mRNA localization is Zipcode binding protein 1 (ZBP1 in chicks, the mouse and human homologues being IMP-1, and in *Xenopus* Vg1RBP). ZBP1 is an RBP important for axonal guidance and branching, and was shown to regulate *β-actin* mRNA localization in axons, which has subsequently provided a seminal example for cue-regulated changes in mRNA localization and translation of a particular mRNA by RBPs [38,39,57,58]. However, in addition to ZBP1, *β-actin* has also been found to directly associate with other RBPs known to be present in axons, such as heterogeneous nuclear (hn)RNP R, and human antigen D (HuD, also known as ELAVL4) [59–61]. As has been shown for ZBP1 binding *β-actin* mRNA in forebrain neuronal axons [35], hnRNP R interacts with the 3′UTR of *β-actin* mRNA to subsequently promote localization to spinal motor axons [61]. Whether hnRNP R directly cooperates with ZBP1 to drive *β-actin* mRNA trafficking in axons, or whether different RBPs regulate axonal localization of the same transcript in different neuron types and contexts remains to be further investigated. However, the finding that hnRNP R knockdown generates a more severe phenotype in motor neuron axons compared to other neuron types [61] suggests that the role of these RBPs in regulating axonal *β-actin* mRNA localization might vary according to neuron type. There are, however, some instances of cooperative regulation by RBPs. For instance, ZBP1 and HuD have been reported to cooperatively regulate axonal localization of *gap-43* mRNA by forming an RNA-dependent complex [62]. Another slightly different example of is the ‘hand over’ mechanism involving ZBP2 and ZBP1. Here, ZBP2 binding of *β-actin* mRNA in the nucleus subsequently facilitates the binding of shuttling ZBP1 that defines cytoplasmic *β-actin* mRNA localization in neurites [63]. Other examples of RBPs directly binding specific mRNAs to regulate their localization in axons include survival of motor neuron (SMN) and nucleolin [64–67], the particular sequences bound by such RBPs are summarized in table 1. In addition to RBP–RNA association, RBP–RBP binding has also been demonstrated to be important for modulating axonal mRNA localization. For example, HuD and SMN were found to interact and colocalize in motor axons where they regulate mRNA localization [80].

**Table 1.** Cis-acting elements identified to be involved in axonal mRNA localization.

mRNA	location of cis-acting element	length (nt)	important features	RBP	reference(s)
annexin A2	CDS (near 3′UTR with 3nt overlap)	18	G-rich motif, primary sequence	SMN	[64,65]
calreticulin and Grp78/BIP	3′UTR	98 and 105	conserved primary sequence	unknown	[68]
cytochrome c oxidase IV (COXIV)	3′UTR	38	predicted hairpin	FUS/TLS, YB-1	[69,70]
Gap43	3′UTR	40	AU-rich regulatory element	HuD–ZBP1 complex	[62]
importin *β*1	3′UTR alternative polyadenylation	34	stem–loop structure	nucleolin	[66,67]
myo-inositol monophosphatase-1 (IMPA1)	3′UTR alternative polyadenylation	120	unknown	unknown	[25]
neuritin/CPG15	3′UTR	40	AU-rich regulatory element	HuD	[71]
synapsin	5′UTR	66	stem–loop structure	unknown	[72]
tau	3′UTR	91	U-rich sequence	HuD, Ilf3 and NF90	[73,74]
Tubb5	3′UTR	37	unknown	unknown	[75]
tyrosine hydroxylase	3′UTR	50	putatively both structure and sequence	unknown	[76]
β-actin	3′UTR	54	primary sequence	ZBP1	[28,77]
β-actin	3′UTR	87	G-rich sequence	APC	[78,79]
*β*2B-tubulin	3′UTR	87	G-rich sequence	APC	[78,79]

While RBPs are important for regulating the export of mRNAs to axons, they can conversely determine whether a particular transcript will be retained in the cell body and excluded from the axonal compartment. This has been observed in cortical neurons, where the RBP telomere repeat-binding factor 2 (TRF2-S) was shown to be involved in regulating axonal trafficking of *rab3a* and *aplp1* mRNAs through binding a glycine–arginine-rich (GAR) domain in TRF2-S [81]. In these neurons, the RBP FMRP was found enriched in the somatic compartment where it competes with *rab3a* and *aplp1* mRNAs for binding to the TRF2-S GAR domain, thus preventing the association of these mRNAs to TRF2-S and reducing the amount of TRF2-S mRNA targets transported into axons. Similar results were also recently obtained in DRG neurons. Here, the RBP Pumilio 2 was observed to be restricted to neuronal cell bodies, where it regulates the axonal transcriptome through somatic retention of a specific subset of transcripts; a process developmentally regulated and essential for proper axonal outgrowth and regeneration [82].

### 3.3. Phase separation and axonal RBPs in mRNA localization

Many recent studies have focused on the assembly of neuronal mRNPs into higher-order granules through liquid–liquid phase separation (LLPS)— a reversible process in which RNAs and associated proteins condense through weak multivalent interactions to separate from the surrounding diluted phase [83,84]. The size, number and molecular components of mRNP granules formed by LLPS can rapidly change depending on the surrounding physical and chemical properties in the cytoplasm [84]. Dynamic rearrangements of mRNP granules have been reported to take place according to specific biological requirements or cellular state (e.g. stress) [84]. For instance, it has been recently reported in *C. elegans* that mRNPs containing the RBP TIAR-2 form liquid-like granules in axons that increase in number following injury *in vivo*; a process that in turn inhibits axon regeneration [85]. The assembly of mRNP granules occurs by a combination of specific protein–protein, protein–RNA and RNA–RNA interactions [86]. RBPs play a key role in promoting liquid-like mRNP formation, and this occurs mostly through domains characteristically composed of only a few different types of amino acids present in high frequency that are referred to as ‘prion-like’, ‘low-complexity’ or ‘intrinsically disordered’ [87]. Various mutations in the low-complexity domain (LCD) of RBPs have been linked to altered mRNP formation in neurons, ultimately inducing aggregates in neuronal compartments and leading to the development of neurodegenerative disorders [88,89]. Some of these LCD containing RBPs, such as TAR DNA-binding protein 43 (TDP43) and fused in sarcoma/translated in liposarcoma (FUS/TLS), have been demonstrated to be involved in axonal mRNA transport and/or translation [90,91]. Unlike membrane-bound organelles, axonal TDP-43 mRNPs have been shown to undergo shape deformation under the shear of fast axonal transport, consistent with surface tension dictating their form [92]. The size and trajectory of axonal TDP-43 mRNPs were also altered following fusion events or transitory interactions, suggesting molecular exchange between mRNP granules [92]. Moreover, TDP-43 mRNPs were found heterogeneous along the axon, exhibiting different granule density and morphology that probably reflect changes in the mRNP composition in different microenvironments [92]. These latter findings raise the intriguing question of how local variation in the levels of RBPs and RNAs (as well as other constituents such as ATP and ions) will modulate mRNP composition along the axon shaft, and thus regulate their trafficking. Remarkably, mRNPs containing TDP-43 mutated in the LCD displayed significant changes in their viscosity to a more solid-like state that correlated with impaired axonal transport [92,93]. These results suggest that axonal trafficking of mRNP granules is dependent on their viscoelastic properties and highlight the importance of the RBP's LCD in this process. A recent study demonstrated that the prion-like domain of the *Drosophila* Imp RBP promotes the axonal motility of Imp-containing mRNPs *in vivo* [94]. Interestingly, the authors also found that the role of the Imp LCD in axonal transport can be uncoupled from its function in mRNP assembly. However, more work will be needed to understand the precise molecular principles underlying mRNP granule assembly by LLPS in neurons, their subsequent axonal transport, and to what extent these granules participate in the precise localization of specific transcripts.

### 3.4. An axonal regulon?

RBPs have been found to target functionally related sets of mRNAs in numerous cell types and organisms. Such an example has been demonstrated in the Puf family of yeast RBPs, where each of the five RBP members binds distinct sets of functionally related mRNAs [95]. In human fibroblasts, mRNAs coding for proteins belonging to the same complex have been reported to colocalize within the same mRNP [96]. Additionally, mRNAs encoding related proteins have been reported to be co-translated and simultaneously assembled into complexes across a range of cell-types and organisms [97]. These findings have led to the idea of ‘RNA regulons’; that to enhance efficiency, groups of mRNAs might be coordinately regulated by RBPs in eukaryotic cells [98]. Since mRNAs residing within the axonal growth cone are enriched with functionally related sets of mRNAs (such as those encoding cytoskeletal-related proteins and proteins involved in mRNA translation [21]), do axonal RBPs also coordinately regulate the localization and translation of these functionally related mRNAs? Recent studies have indeed suggested that this might be the case. Adenomatous polyposis coli (APC) is a microtubule-associated protein that is enriched at the axon tip where it plays roles in axon growth, navigation and morphology by regulating the cytoskeleton [99–102]. Excitingly, this protein has also been shown to additionally function as an RBP in axons [78]. Here, APC was discovered to bind multiple mRNAs related to its known function as a cytoskeletal regulator (e.g. mRNAs encoding microtubule-associated motors and tubulin isotypes). Of these APC-associated mRNAs, *β2B-tubulin* mRNA was found to rely on APC for its axonal localization and translation, a regulation required for proper cytoskeletal dynamics and growth cone morphology [78]. More recently, the RBP splicing factor poly-glutamine rich (SFPQ) was shown to orchestrate the axonal transport of mRNAs coding for survival factors essential to maintain DRG axons [103]. In addition, SFPQ was found to promote the co-assembly of these functionally linked mRNAs within the same mRNPs; a role that could directly support their synchronized axonal translation when required [103].

That axonal regulons could provide an efficient means to coordinately regulate sets of mRNAs to carry out specific functions raises the question of whether these mRNAs are transported together or individually. Studies looking at mRNAs localized in neuronal dendrites have revealed that dendritic mRNAs travel in low copy numbers and that certain different mRNA species are transported independently [104–106]. Similarly, within axons, mRNAs have been shown to mostly travel in low copy numbers [107] while some mRNAs, but not others, have been reported to co-assemble in the same axonal mRNP [103]. Since one mRNA can be translated multiple times, transporting lower copies of mRNAs may allow a tighter control over the quantity and localization of axonal mRNAs. However, stoichiometry analysis has only been performed for a limited number of mRNA species, and it remains conceivable that mRNA transport could be more multiplexed for certain mRNA species or circumstances (e.g. as observed for some germ plasm mRNAs in *Drosophila* [108]). Indeed, this is a question that is particularly intriguing in the case of mRNPs formed by LLPS.

In conclusion, while many more questions still need to be answered regarding the role and mode of action of individual RBPs, proper axonal trafficking of a particular mRNA appears to rely on a combination of RBP–RNA and RBP–RBP bindings that are dynamically modulated depending on the functional needs of the axon.

## 4. mRNA features that drive their axonal localization

mRNA recognition by specific RBPs and their subsequent localization patterns pivot on particular structural and primary sequence features within the mRNA itself. These ‘cis-acting elements’, or ‘zipcodes’, have been shown to control the mRNA's transport and anchoring in subcellular compartments [109].

### 4.1. Cis-acting elements that drive axonal mRNA targeting

Cis-acting elements come in many shapes and sizes, ranging from just a few nucleotides to hundreds of nucleotides in length [109]. Relatively few mRNA cis-acting elements and corresponding RBPs have been identified for axonal localization (table 1). Just like the cis-acting elements involved in mRNA localization within other cell types [109], these can depend on nucleotide sequence, structure, or both, and are mostly found in the 3′UTR. One example is the axonal localization of cytochrome *c* oxidase IV (COXIV) mRNA, a nuclear-encoded mitochondria protein involved in oxidative phosphorylation, where its local translation is important for ATP production and axon growth [69,110]. Here, a 38-nucleotide predicted stem–loop structure in COXIV mRNA was found sufficient to confer axonal mRNA localization in superior cervical ganglion neurons [69]. Immunoprecipitation of the COXIV mRNA cis-acting element revealed association with approximately 53 proteins, forming a distinct interactome compared to those of neuronal mRNP granules associated with the RBPs Staufen2 and Barentsz (two RBPs associated with mRNP transport to dendritic neuronal compartments) [70,111]. Of these COXIV mRNA cis-element interacting proteins, the RBPs FUS and Y-box protein 1 (YB-1) were further validated as *bona fide* binding partners, and their knockdown led to significantly decreased levels of COXIV mRNA in axons [70]. Here, while affecting axonal COXIV mRNA levels, FUS siRNA treatment did not affect cell body levels of COXIV mRNA, suggesting that this RBP might specifically regulate mRNA localization to axonal compartments rather than affecting mRNA stability or earlier stages like transcription [70].

**Figure 2.**
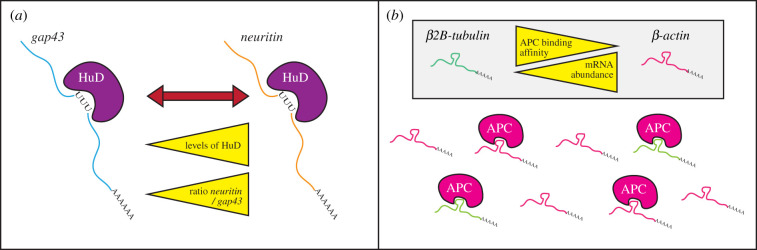
Cis-acting elements regulate mRNA localization in axons. (*a*) Differential binding affinities to cis-acting elements regulates RNA binding by HuD. In motor neurons, *gap43* mRNA AU-rich element has a higher binding affinity to HuD than the *neuritin* mRNA AU-rich element, which thus regulates *neuritin* mRNA binding through competition. Increased HuD and decreased *gap43* mRNA levels subsequently permit HuD-dependent axonal localization of *neuritin*. (*b*) Higher binding affinity of APC to G-rich sequences in *β2B-tubulin* mRNA 3′UTR compared G-rich sequences in the *β-actin* mRNA 3′UTR are proposed to ensure that less abundant mRNAs are transported.

Another example of mRNA cis-acting elements in axons are the short AU-rich elements in the mRNAs encoding Gap-43, Neuritin and Tau, which are all recognized by the RBP HuD [62,71,73]. Each of these AU-rich cis-acting elements targeted by HuD are capable of driving mRNA localization to axons [62,71,73]. Two RRM domains of HuD have been shown to directly bind cis-acting elements in the mRNA, while the third binds the mRNA's poly(A) tail, which stabilizes that mRNP complex [112]. HuD has known roles in mRNA stability [113], and has been demonstrated to stabilize *neuritin* mRNA in the brain [71], raising the question as to whether these differences in localization might be partly due to increasing mRNA lifetime in axons. Intriguingly, the differential binding affinity of cis-acting elements to HuD appears to be a key factor for HuD-dependent regulation of mRNA metabolism. *gap43* mRNA has a higher binding affinity to HuD than *neuritin* mRNA, resulting in competition-dependent regulation of axonal mRNA localization [71]. Recently, the RNA target with the highest affinity for HuD binding in differentiating motor neuron cultures was shown to be a small noncoding RNA Y3 [114]. Y3 acts as a ‘molecular sponge’ to sequester HuD away from polysomes and supress mRNA translation needed for neural differentiation in that system [114]. These findings hint at the complexity of mRNA regulation by cis-acting element recognition of HuD—involving an interplay of differential cis-element binding affinities (figure 2*a*). Interestingly, different cis-element binding affinities were also recently suggested to act as a mechanism to regulate the amount of mRNA species transported in the axon [79]. Here, the RBP APC (which has been demonstrated to regulate axonal mRNA localization [78]) was shown to selectively bind G-rich sequences in the 3′UTR of *β2B-tubulin* and *β-actin* mRNAs to enable their trafficking [79]. In cortical and hippocampal neurons, *β-actin* mRNA is in greater than or equal to 10-fold greater abundance than *β2B-tubulin* mRNA [115], but *β2B-tubulin* mRNA has a fivefold higher affinity for binding to APC [79]. These differential binding affinities were thus intriguingly suggested to act as a mechanism to ensure that less abundant mRNAs are still transported into axons (figure 2*b*). Axonal mRNA localization is, however, not regulated by one type of RBP and corresponding cis-acting element. Interestingly, a recent study revealed that no one RBP interaction is shared by four different cis-acting elements (belonging to four different mRNAs) that can each drive axonal mRNA localization in motor neurons [116]. Instead, it was discovered that particular hnRNPs bind one or two different cis-acting elements, indicating that different combinations of RBPs coordinately regulate axonal mRNA transport through different RNA localization signals in axons [116]. As a result, one of the most exciting questions for future studies will be to further understand how different RBPs work together with differences in cis-element binding affinities to fine-tune mRNA levels in axons.

### 4.2. The axon–dendrite paradox

Despite a neuron's dendrites and axon being structurally and functionally distinct, many of the identified axonal mRNA cis-acting elements also paradoxically drive mRNA localization to dendrites. This raises the crucial question of how subcellular localization specificity is achieved in neurons through the same cis-acting element. One well-known example is the zipcode region of *β-actin* mRNA. In addition to its role in regulating axonal mRNA localization, it also targets mRNAs to dendrites of neurons, as well as the leading edge of fibroblasts [77,117,118]. The zipcode cis-acting element in *β-actin* mRNA is recognized by the RBP ZBP1 [77]. ZBP1-dependent localization of *β-actin* mRNA relies on a bipartite 54 nucleotide ‘zipcode sequence’ in the 3′UTR of *β-actin* mRNA, and is required for the increased localization of the mRNA in axonal growth cones in response to particular cues [35,38,119,120]. Other examples include two cis-acting elements in the 3′UTR of *calreticulin* mRNA, which drive both dendritic and axonal mRNA localization within the same cortical neurons [68]. Furthermore, many RBPs that regulate axonal mRNA localization, like ZBP1, FMRP, HuD and FUS, are also localized to dendrites and implicated in regulating dendritic mRNA localization [118,121–123]. Dendrites have different functions compared to axons, respond to different signals and additionally contain different transcriptomes (where for example *map2* mRNA is enriched in dendrites but excluded from axons [27,124]). If they often have the same cis-acting elements, bound by the same RBPs, how then might these sub-compartmental mRNA localization patterns be differentially regulated in neurons? One possibility is that many of the identified cis-acting elements and associated RBPs might regulate more general cytoplasmic localization in neurites, while additional cis-acting elements and RBPs are required for sorting into different neuronal compartments. For instance, *synapsin* mRNA contains two cis-acting elements: one in the 5′UTR and one in the 3′UTR [125]. Here, the 3′UTR of *synapsin* mRNA is able to confer localization to distal neurites, but only a 66 nucleotide stem–loop structure in the 5′UTR is able to localize the mRNA to the synapse [72,125]. Indeed, a similar concept is also seen during *Xenopus* and *Drosophila* germ plasm development. Here, different cis-acting elements target mRNAs at different stages of the localization process [126–129].

While specific axonal mRNA targeting signals remain poorly understood, in the reverse direction of inquiry, a cis-acting element was recently identified to keep mRNA out of axons. Here, Martínez et al. [82] employed a bioinformatics approach to look at sequences associated with the axonal versus somatodendritic transcriptome. Although the authors found no sequence motifs associated with the axonal transcriptome, a UGUAAAU motif was discovered as enriched within the somatodendritic transcriptome. When inserted into ordinarily axonally localized transcripts this motif was able to diminish axonal localization; the RBP responsible was identified to be Pumilio 2 [82]. In the future, new techniques, such as ‘proximity-CLIP’, [130] might be able to help us further decipher how differences in protein–RNA interactions lead to altered mRNA localization patterns between axons, dendrites and soma.

## 5. Lights, camera, axon! Unravelling the mechanisms behind mRNA movement in axons

Understanding how mRNAs are trafficked in axons will solve a large piece of the puzzle elucidating how specific axonal mRNA localization patterns are generated. Often deciphering how mRNA molecules move involves the use of live-imaging methods, the techniques and advances for which are described in box 1. In this section, we will provide a general overview of mRNA transport mechanisms, before delving into what is currently known about mRNA transport in axons.

Box 1. **Live-imaging approaches to study mRNA transport.**

Understanding the type of motion that takes place in cells often necessitates live-imaging approaches to directly visualize mRNA movement. To this end, there have been several methods developed, each with their own advantages and caveats.

Firstly, one could image exogenous mRNA by *in vitro* transcribing the mRNA of interest using UTP-conjugated organic fluorophores and subsequently deliver this fluorescently labelled synthetic mRNA into cells [131]. The main advantage of this approach is the specificity, high signal-to-noise ratio and speed of experimental design. However, exogenous mRNA can be delivered into the cytoplasm above physiological levels and proper mRNA localization often involves nuclear processing events [132], thus exogenous mRNA may not always mimic the same transport and localization methods as their endogenous counterparts. Nonetheless, the delivery of exogenous mRNA has been demonstrated to recapitulate endogenous localization patterns in several systems and is a particularly useful approach for analysing sequence and mRNA processing requirements for trafficking and localization [133–135]. One of the most popular methods for studying endogenous mRNA trafficking is the MS2 system [136,137] (as well as variants such as the PP7 system [138]). The MS2 approach involves genetically engineering your mRNA of interest to encode multiple MS2 hairpins each of which are recognized and bound by two MCPs (MS2 coat proteins) fused to a fluorescent protein. This method has been prototypical for imaging endogenous mRNAs and led to many exciting discoveries. However, false positives can arise from non-specific conjugation of MCP-fluorescent proteins [139] and the formation of decay fragments [140]. Many advancements of the MS2 system have been published (for example increasing signal-to-noise [141] and allowing life cycle visualization [142]). Recently, modifications of the CRISPR/Cas9/13 systems have provided an exciting alternative to the MS2 approach, enabling labelling of endogenous RNA without time-consuming genetic manipulation, albeit with lower signal to noise [143,144]. In addition, fluorogenic RNA aptamers provide a method for live mRNA imaging by inserting the aptamer into the mRNA of interest. This approach offers the advantage of lower background signal and since the development of Spinach RNA aptamer that exhibits fluorescence resembling GFP [145], different advancements have been made to include increased photostability and brightness [146] as well as fluorescence at different wavelengths [147]. Finally, another alternative approach is to use molecular beacons [148,149]. Here, each molecular beacon is designed to contain an antisense probe region that binds to the endogenous mRNA of interest, as well as a GC-rich stem region that places a fluorophore and quencher in close proximity. Binding of the antisense probe region to the mRNA changes the molecular beacon conformation so that the fluorophore is separated from the quencher, thus labelling the endogenous mRNA of interest with fluorescence. While this approach is experimentally simple and allows visualization of endogenous mRNA without genetic modification, non-specific signal in the nucleus as well as non-specific background labelling necessitates the use of good controls.

To our knowledge, only molecular beacons and fluorescent exogenous mRNA have been used to study mRNA transport in axons [37,93,107]. We anticipate, however, the use of different techniques to study axonal mRNA transport in the future, in addition to new data analysis techniques such as automated kymograph analysis by machine-learning [150].

### 5.1. A short introduction to mRNA transport in cells

In general, there are three main mechanisms by which mRNAs move in cells: directed transport, diffusion or a mixture of both [151]. Diffusion describes the Brownian motion of molecules due to fluctuations in thermal energy, creating a random walk in which the molecule has an equal propensity to move in any direction [152]. Without other mechanisms to direct localization, diffusion will eventually create a uniform spread of molecules across the cytoplasm. This distribution is seen for several non-localizing mRNAs [153], although even uniformly distributed mRNAs appear to undergo some periods of directed transport [154]. Asymmetric localization can be achieved by diffusion through external factors that bias random movement in one direction and/or locally anchor diffusing mRNAs. An example for both mechanisms is illustrated by *Drosophila nanos* mRNA that is localized to the posterior of the oocyte via a ‘diffusion and entrapment mechanism’ involving a combination of ooplasmic streaming and local anchoring at the posterior pole [155,156].

Perhaps one of the most common mechanisms for achieving targeted mRNA localization is through directed transport by motor proteins. Directed transport involves motor proteins that use ATP hydrolysis to trigger conformational changes that generate processive movement along microtubule or actin microfilament tracks. The myosin superfamily of motor proteins associate with actin filaments, whereas kinesin and dynein families of motor proteins travel along microtubules in opposing directions (kinesins towards plus ends of microtubule polymers and dynein towards minus ends) [157]. Since polarized cytoskeletal organization is often a common feature among polarized cells [158], preferential association with specific motor proteins promotes directed transport to particular cellular regions. Many examples for directed transport by motor proteins exist for mRNA localization. For instance, in budding yeast, the type V myosin motor is required for *ASH1* mRNA localization [159,160], and the concerted actions of the microtubule-associated Kinesin1 and Kinesin2 motor proteins are needed for *Vg1* mRNA targeting to the vegetal cortex of *Xenopus* oocytes [161]. In some instances, mRNAs can bind opposing motors, resulting in bidirectional movement [162]. Here, asymmetric localization is achieved by biasing motion in one direction through, for instance, increasing the copy number of a specific motor protein. For example, mRNA cis-acting elements have been directly demonstrated to alter dynein copy number to drive biased bidirectional movement and apical localization of the *Drosophila fs(1)K10* mRNA transcript [163]. In addition, bidirectional directed transport can be coupled with local anchoring. This has been observed for the local entrapment of *β-actin* mRNA at activated synapses in dendrites [164]. Given these now well-established mRNA transport methods in different cell types and model systems, how are mRNAs transported into the axon of neurons?

### 5.2. Microtubule-driven mRNA transport in axons

**Figure 3.**
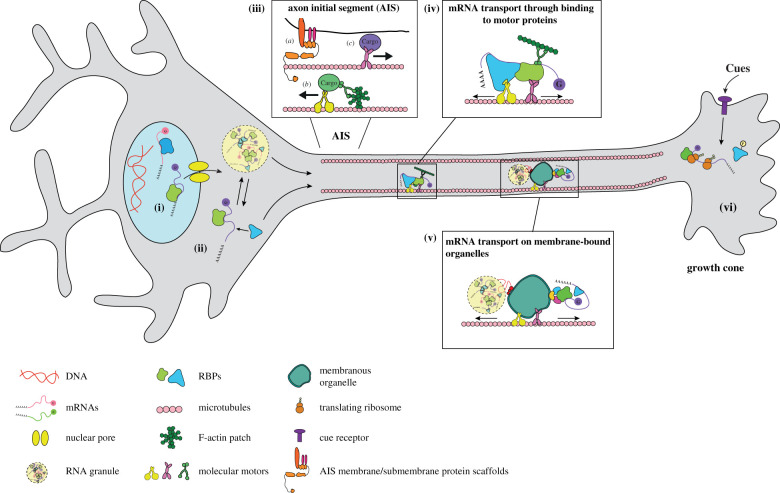
Model for mRNA localization in axons. (i) mRNP formation: mRNAs are initially recognized by RBPs in the nucleus co- and post-transcriptionally. (ii) mRNAs are transported into the cytoplasm where mRNP remodelling occurs. (iii) Somatodendritic and axonal cargo are sorted at the axon initial segment (AIS): (*a*) membrane proteins linked to the actin cytoskeleton form a diffusion barrier at the AIS, inhibiting free diffusion of molecules. (*b*) Somatodendritic proteins do not enter the axon shaft but are instead immobilized by Myosin V at F-actin patches and return to the soma by dynein motor protein driven transport. (*c*) Proteins destined to axons are associated with kinesin motor proteins, which are thought to recognize signals on microtubules and are trafficked into the axon (such a mechanism is likely to also be involved in axonal versus dendritic localization of mRNAs in neurons). (iv,v) mRNAs associated with RBPs travel along polarized microtubule cytoskeletal tracks. mRNPs are transported by motor proteins via (iv) a cargo adaptor protein or (v) by hitchhiking on membranous organelles. (vi) mRNAs dissociate from RBPs at sites of local translation.

The sheer length of neuronal axons logically suggests that directed transport is important. Indeed, live-imaging has shown that *β-actin* mRNA exhibits directed movement in axons as well as periods of diffusion; the directed transport component being essential for mRNA delivery to the axon tip [107]. Since the discovery that mRNA does not accumulate in nerve terminals following disruption of microtubules by colchicine [165], numerous studies have demonstrated that basal and stimuli induced changes in axonal mRNA localization rely on an intact microtubule cytoskeleton [31,35,166,167]. Further, direct visualization of exogenous fluorescently tagged *β-actin* mRNA movement recently revealed that the integrity of the microtubule cytoskeleton is essential to maintain directed transport [37]. As such, it is clear that microtubule-driven directed transport is important for localizing mRNA to the axon tip and various underlying molecular mechanisms have now been proposed, leading to a generalized hypothetical model for mRNA trafficking in axons (figure 3).

Different motor proteins have different kinetic properties and polarity preferences that in turn can generate unique cellular distributions for different cargoes [168]. What are the motor proteins that specifically transport mRNAs in axons? In axons, microtubule orientation is highly polarized. Greater than 90% of microtubules are orientated plus-end out, with growing ends pointing towards the axon tip [169]. In addition, different microtubule-associated motor proteins drive either plus-end or minus-end directed motion (kinesin and dynein towards plus and minus ends, respectively) [157]. Thus, axonal anterograde microtubule-driven transport requires the kinesin motor protein family, while retrograde microtubule transport is driven by cytoplasmic dynein. Since mRNA movement in axons is bidirectional [37,93,107,170,171], it follows that both should play a role. Indeed, bidirectional transport of other axonal cargoes has been shown to involve the simultaneous association of opposing kinesin and dynein motors that cooperatively regulate processivity [172]. While cytoplasmic dynein is the major retrograde microtubule motor transporting cargo in axons, multiple kinesin motor proteins perform axonal anterograde transport; at least 3 of the 14 kinesin subfamilies (kinesin-1, kinesin-2, kinesin-3) are reported to transport axonal cargos [173]. Different kinesins have been shown to possess different kinetic properties and transport distinct cargo, as well as in some instances cooperatively transport the same cargo [169,174]. Only recently have specific kinesin motor proteins been associated with mRNA transport in axons. Axonal mRNA transport of APC has been shown to depend on kinesin-1 and kinesin-2 [175] and APC-dependent transport of *β-actin* and *β2B-tubulin* mRNAs was recently demonstrated to involve APC complexed with kinesin-2, via the KAP3 cargo adaptor protein within reconstituted complexes *in vitro* [79]. Although it will be important to ascertain whether APC transport driven by kinesin-2 also regulates mRNA trafficking within neuronal axons themselves, these data strongly support a role for kinesin-2 in axonal mRNA transport by APC. Moreover, that interactions with both kinesin-1 and kinesin-2 regulates localization of the RBP APC to axon tips [175], and that axonal *β-actin* mRNA transport exhibits multimodal velocities [107], are indicative that multiple motors and/or regulator proteins might be at play. In another study, Fukuda *et al.* [176] provided direct evidence for particular kinesins driving axonal mRNA transport by another RBP. Here, the authors excitingly found that the RBP SFPQ interacts with the heavy chain kinesin-1 family member KIF5A via the adaptor KLC1; this binding was shown to take place in an RNA dependent manner and is needed for driving axonal transport of SFPQ [176]. That these two studies identified different kinesin family members involved in transporting different axonally localized RBPs raises the fascinating question of how RBPs and their associated mRNA interactomes might differentially associate with different motor proteins to uniquely spatiotemporally regulate axonal mRNA localization. Do, for example, different RBPs exhibit distinct kinetics in axons that might be indicative of different transport mechanisms? Moreover, do different motor proteins play separate roles in the transport of the same mRNA depending on the neuron-type and/or developmental stage?

### 5.3. A role for actin in axonal mRNA localization?

Although plentiful evidence suggests microtubules are important in axonal mRNA trafficking, coordination with motors associated with the actin cytoskeleton has also been implicated. Here, cue-stimulated increases in axonal mRNA localization are slightly affected by actin filament disruption [31]. Similarly, under basal conditions actin depolymerization results in failure of microinjected BC1 mRNA to concentrate in cortical axonal domains, leading to the suggestion that myosin-driven motor transport might play a role in short-range axonal mRNA transport [166]. By contrast, recent work has shown that actin filaments are not needed for cue-induced changes in exogenous *β-actin* mRNA to peripheral domains of RGC axon growth cones [37], but appears to play a role in anchoring endogenous *β-actin* in the same axons [107]. The actin cytoskeleton is also fundamental for axonal cargo sorting. Cargo sorting in axons involves the axon initial segment (AIS), which prevents entry of somatodendritic cargo (figure 3). This occurs firstly by anchoring a crowd of membrane proteins to provide a diffusion barrier [177]. Secondly, by actin patches within the axon hillock that immobilize somatodendritic cargo by myosin V association with particular motifs on somatodendritic proteins [178]. Thirdly, through axon cargo association with particular microtubule-associated motor proteins that direct axonal trafficking due to the polarized microtubule orientation in axons [179]. It is likely, therefore, that axonal mRNA localization might similarly involve a sorting mechanism in the AIS triggered by (as yet unidentified) cis-acting elements and RBPs. In support of this, both actin filament depolymerization and inhibiting myosin Va leads to axonal localization of the normally somatodendritic cargo *map2* mRNA and Staufen 1 RBP [180].

### 5.4. Getting there by organelle hitchhiking in axons

Recent data suggest that a certain number of mRNPs can travel along the cytoskeleton by hitchhiking on intracellular organelles in axons (figure 3). For example, mRNP granules, (probably assembled by LLPS) have been found to be co-transported with lysosomes in cortical axons [181]. Here, similar to observations of a lipid-binding adaptor protein allowing RBP and mRNA recruitment on endosomes in fungal hyphae [182], the annexin protein ANXA11 was shown to couple RNA granules to lysosomal compartments [181]. ANXA11 has several specific features that allow this function, including a prion-like domain that facilitates its association with mRNP granules and a Ca^2+^-dependent lipid-binding domain that regulates its interaction with lysosomes. While intriguing, the exact function for this type of transport in regulating the axonal transcriptome is not yet clear, but the fact that an association between a portion of mRNPs and endosomal compartments has been reported in *Xenopus* RGC axons [170] supports a potential more broad role for membrane trafficking in axonal mRNA localization. In both *Xenopus* axons and fungal hyphae, RNA granules associated with endosomes displayed bidirectional movement, and were found to associate and dissociate from endosomal compartments—a feature that could ensure the proper distribution of specific transcripts [170,183]. Evidence in both systems also point toward the idea that some mRNAs are translated on endosomes [170,183,184], adding a complementary role for endosomes as specific sites of protein synthesis. This dual function is further supported by a recent study showing colocalization between early endosomes and specific mRNAs in a HeLa cell line; an association relying on intact ribosomes for a significant proportion of mRNAs, indicating that these mRNAs localize to endosomes in a translation-dependent fashion [185]. In this system, the spatial distribution of specific transcripts was found altered upon manipulation of early endosome trafficking, thus suggesting co-transportation of endosomes and mRNAs. As other organelles present in axons (e.g. mitochondria, endoplasmic reticulum, peroxisomes) have also been shown to harbour mRNAs on their membrane [171,186–188], these results open the possibility that hitchhiking on membranes could be an important regulatory process in axonal mRNA trafficking.

### 5.5. Regulation of axonal mRNA localization by degradation

Finally, although not a transport mechanism, mRNA decay has been shown to be an important way by which mRNA localization patterns are generated for particular mRNAs and cell types. One classic example is posterior localization of *hsp83* mRNA (encoding a heat shock protein) in the *Drosophila* embryo generated by selective protection from degradation at the posterior pole of the embryo that is regulated by cis-acting elements in *hsp83* 3′UTR as well as cis-acting elements in the open reading frame generating increased destabilization in the rest of the cytoplasm [189–192]. Nonsense-mediated decay (NMD) is a degradation pathway, which usually ensures that improperly translated mRNAs (carrying premature termination codons) are degraded [193]. In commissural neuron axons, this method of degradation is used to regulate *Robo3.2* mRNA levels (encoding an axon guidance receptor) after *Robo3.2* translation is derepressed at the floorplate [194]. As well as commissural axons, axonal growth cones of different neuron-types (dorsal root ganglia and hippocampal neurons) are also enriched with components of NMD machinery, suggesting that local regulation of mRNA degradation in the growth cone may be a common method for regulating mRNA localization and translation at the axon tip [194]. Because NMD targets particular transcript features (*Robo3.2* for example has a retained intron [194]) axonal NMD degradation is likely to selectively regulate a subset of mRNA transcripts. At the other end of the table, recent work has suggested that certain mRNAs are protected from degradation in axons [195]. Here, Nikolaou *et al.* [195] revealed that the major spliceosome protein SNRNP70 associates with mRNAs in axons and protect certain transcripts from NMD-mediated degradation through alternative splicing [195]. How degradation interplays with mRNA trafficking mechanisms to spatiotemporally regulate mRNA localization in axons will be an exciting question for future studies that will be enabled through methods to visualize mRNA degradation such as TREAT [196].

## 6. When axonal mRNA localization mechanisms go wrong

Defects in axonal mRNA localization have been associated with an increasing number of neurological disorders affecting different areas of the brain. These disorders with links to disrupted axonal mRNA localization are summarized in table 2 and further expanded upon below.

**Table 2.** Neurological disorders linked to impaired axonal mRNA localization.

disease name	experimental model	associated impairment	reference(s)
fragile X syndrome (FXS)	knockdown of FMRP in primary mouse embryonic DRG neurons	reduced axonal transport of *miR-181d*, *Map1b* and *Calm1*-positive granules	[197]
Alzheimer's disease (AD)	treatment of rat embryonic hippocampal neurons distal axons with oligomeric A*β*_1–42_	altered axonal transcriptome with 151 upregulated and 211 downregulated transcripts, including the relevant *atf4* mRNA	[198]
amyotrophic lateral sclerosis (ALS)	compartmentalized cultures of SOD1^G93A^ and TDP43^A315T^ mouse embryonic motor neurons	altered axonal transcriptomes with 176 upregulated and 271 downregulated transcripts in TDP43^A315T^ axons compared to controls, and 95 upregulated and 80 downregulated transcripts in SOD1^G93A^ axons compared to controls; both models show an increase of the mRNA coding for the RBP Elavl2 in axons	[30]
amyotrophic lateral sclerosis (ALS)	*Drosophila*- and human-derived motor neurons bearing ALS-causing mutations in TDP43	*Nefl* mRNP granules anterograde axonal transport is significantly impaired after 9 days in culture	[93]
amyotrophic lateral sclerosis (ALS)	mouse embryonic stem cells (mESCs)-derived motor neurons overexpressing SOD1^G93A^	altered axonal transcriptome with 96 upregulated and 25 downregulated transcripts between mutated and control axons	[199]
spinal muscular atrophy (SMA)	knock-down of survival motor neuron (SMN) protein in mouse embryonic motor neurons (shRNA)	general reduction of polyA-positive RNAs in the axonal compartment evaluated with qFISH using digoxigenin-labelled oligo-dT probes	[80]
spinal muscular atrophy (SMA)	knock-down of survival motor neuron (SMN) protein in mouse embryonic motor neurons (shRNA)	altered axonal transcriptome with 165 upregulated and 1189 downregulated transcripts in axons depleted for SMN compared to controls	[200]

### 6.1. Neurodevelopmental disorders

Although the functional importance of axonal mRNA translation has mostly been demonstrated in developing neurons, only a restricted number of developmental disorders have been linked to altered mRNA transport. One of these is fragile X syndrome (FXS), an X-linked disease caused by the loss of the RBP FMRP [201]. It has been shown that FMRP has the ability to associate with a vast number of mRNAs [202,203], and multiple mechanisms underlying its role in controlling mRNA translation have been described [204–206]. However, less is known regarding FMRP function in mRNA trafficking. FMRP was found to be present in developing [207] and mature [208] distal axons, and loss of its function has been associated with defects in growth cone dynamics and axon growth [207,209]. In mouse DRG axons, FMRP has been shown to associate with *map1b* and *calm1* mRNAs, and its depletion was found to impair axonal mRNA targeting [197], supporting a role for FMRP in axonal mRNA localization (albeit a role that has been suggested to be more complicated by other work [81]). In a recent study, subcellular fractionation and high-throughput sequencing were applied in a mouse neuronal tumour line (CAD) to identify the transcripts differentially localized in somata and neurites in the absence of FMRP [210]. The authors found a large number of neurite-depleted mRNAs in the absence of FMRP, a result that they further confirmed using iPSC-derived motor neurons from FXS patients. They also demonstrated that FMRP regulates the transport of transcripts containing G-quadruplex motifs in their 3′UTR through the binding to its RGG domain. Intriguingly, the function of FMRP in mRNA localization appeared dissociated from its role in translational regulation, which is potentially achieved through different modes of target recognition. Although there has been some light shed on the physiological function of FMRP and the defects caused by its depletion, the direct functional consequences of mislocalized mRNAs in axons in FXS pathogenesis still needs to be determined.

### 6.2. Neurodegenerative disorders

In recent years, the potential link between impaired axonal mRNA localization and neurodegenerative disorders have received increasing attention. This is particularly the case for amyotrophic lateral sclerosis (ALS), a multigenic disease resulting from mutations in various genes including superoxide dismutase (SOD1), TDP43 or FUS protein [211]. Expression of ALS-linked mutated forms of FUS were found to decrease axonal protein synthesis in *Xenopus* RGC axons [212]. Moreover, expression of FUS mutants at concentrations close to physiological levels of endogenous FUS, leads to the accumulation of the mutant protein along the axons of hippocampal neurons *in vitro* and sciatic nerves *in vivo*, and is sufficient to cause a reduction in intra-axonal protein synthesis, in turn leading to motor and cognitive impairment [213]. Although a direct link with impaired axonal mRNA transport is missing, it has recently been shown that, upon mutation of FUS and formation of FUS-positive inclusions, APC-dependent transport of mRNAs to cell protrusions is disrupted, both in neurons and non-neuronal cell types [214], supporting a direct role in mRNA trafficking in neuronal compartments.

As described in a previous section of this review, TDP43 is an RBP that participates in the assembly of mRNPs [215]. While accumulation of TDP43 is observed in frontotemporal lobar degeneration (FTLD) [216], its role in axonal mRNA trafficking has been shown to be impaired by several ALS-causing mutations of the protein. In particular, trafficking of TDP43 along cortical axons was found altered by mutations in its prion-like C-terminal domain, possibly by affecting phase separation and mRNP formation [93]. These ALS-causing mutations were also found to compromise axonal trafficking of Neurofilament-L (*Nefl*) mRNA *in vivo* in *Drosophila*, and in mouse cortical neurons [93], supporting a direct link between ALS-associated mutations of TDP43 and defects in axonal mRNA transport. Since then, hundreds of mRNAs have been shown to be differentially present in axons isolated from TDP43^A315T^ murine motor neurons [30]. Interestingly, the same study compared these changes to the alterations induced by the ALS-associated mutation SOD^G93A^. While mutations in these two genes lead to distinct direct functional consequences, they found common altered axonal levels of specific transcripts, supporting the hypothesis that ALS might be initiated by defective mRNA localization in axons of particular neuron types.

Interestingly, similar observations have been reported in neurons carrying mutations associated with spinal muscular atrophy (SMA). SMA is a neuromuscular disease characterized by the degeneration of spinal motor neurons and caused by reduced levels of the RBP SMN due to deletions or, less frequently, mutations in the SMN1 gene [217,218]. Several observations have led to the hypothesis that SMN protein might be important for localizing a subset of important mRNAs to the neuromuscular junction (NMJ). Firstly, SMN is transported into motor neuron axons and knockdown of the protein results in a reduction of both poly(A) mRNA levels and HuD protein present in axons [80]. Secondly, analysis of the corresponding changes in the axonal transcriptome following SMN knockdown revealed decreased levels of mRNAs encoding proteins with roles in axonal growth and synapse regulation [200], supporting an important role in presynaptic function. Interestingly, when compared to the changes observed following SOD^G93A^ overexpression [199], 16 genes were identified as commonly dysregulated, suggesting that some common pathological-associated changes in axonal mRNA localization exist between different neurodegenerative diseases.

While less studied, pathologies affecting the central nervous system have also been found associated with altered axonal mRNA repertoires. Alzheimer's disease (AD) is a neurodegenerative disorder affecting diverse brain areas and mainly characterized by the accumulation of β-amyloid (A*β*) in neurites in particular soluble oligomers of A*β*_1–42_ [219–221]. Application of A*β*_1–42_ to distal axons of rat embryonic hippocampal neurons was reported to induce changes in the local transcriptome, including mRNAs related to A*β* production and metabolism, such as *app*, *apoE* and *clu*, or involved in tau pathology like *fermt2* [198]. The authors further focused on *atf4* mRNA which was found recruited to the axon upon treatment with A*β*_1–42_, where its intra-axonal translation was suggested to participate in causing neurodegeneration linked to AD.

An increasing number of observations are now pointing towards the hypothesis that alterations in the axonal transcriptome could participate in the pathophysiology of various diseases, including disorders that are not directly associated with mutations in genes coding for well-known regulators of RNA processing. While more work will be needed to clearly demonstrate the therapeutic potential of counteracting defects in axonal mRNA mislocalization, an interesting example supporting this possibility was recently reported. Here, mutations in the gene coding for the motor protein KIF5A have been linked to Charcot–Marie–Tooth disease type 2D (CMT2D), amyotrophic lateral sclerosis (ALS) and hereditary spastic paraplegia (HSP) [222–224]. KIF5A was found important for the axonal localization of SFPQ mRNPs in DRG axons [176]; an RBP that has previously been shown to control a regulon of transcripts coding for survival factors, including regulating the axonal trafficking of the mRNA encoding Bclw [103]. Remarkably, the authors also found that axon degeneration induced by CMT2D KIF5A mutation can be rescued by introducing a peptide that mimics the function of the axonally synthesized protein Bclw [176]. This approach could potentially be extended to other diseases with defects in axonal mRNA transport and/or translation. Moreover, that mRNAs have been found associated with various organelles, including endosomes and lysosomes in axons [170,181], also opens new therapeutic perspectives for diseases in which organelle transport has been disrupted.

## 7. Conclusion

It is now unambiguously established that mRNA translation in axons is important for neuronal development, function and survival, but our current knowledge of how specific mRNAs become localized to the axonal compartment in the first place remains limited. As illustrated in this review, some advances have been made in the field, including the identification of key cis-acting elements and trans-acting factors involved in axonal mRNA targeting and transport. However, many important open questions still remain. For example, it will be important to increase our repertoire of sequence elements, and associated RBPs, involved in the ‘positive’ or ‘negative’ targeting of specific mRNAs to the axon. With respect to cis-acting sequences, further understanding the role of secondary and tertiary mRNA structures will be crucial to both identifying new cis-acting elements as well as further understanding the mechanisms by which sequences already identified act. Moreover, it will also be important to understand how the cooperative actions of different RBPs and motor machinery components fine-tune the localization patterns of multiple different mRNAs in axons. Finally, future work will be needed to explore the functional relevance of axonal mRNA localization *in vivo*. For instance, how is the dynamics of axonal mRNA transport affected by synaptic activity? Is this process experience dependent? What would be the consequences of impairing axonal trafficking of particular subsets of mRNAs on brain function? As a strong link between neurological diseases and defective axonal mRNA localization is currently emerging, the answer to these issues might be key to better understand the pathophysiological mechanisms behind these disorders.

## Data accessibility

This article has no additional data.
